# Soft pneumatic muscles for post-stroke lower limb ankle rehabilitation: leveraging the potential of soft robotics to optimize functional outcomes

**DOI:** 10.3389/fbioe.2023.1251879

**Published:** 2023-09-14

**Authors:** Mostafa Orban, Kai Guo, Hongbo Yang, Xuhui Hu, Mohamed Hassaan, Mahmoud Elsamanty

**Affiliations:** ^1^ School of Biomedical Engineering (Suzhou), Division of Life Sciences and Medicine, University of Science and Technology of China, Hefei, China; ^2^ Suzhou Institute of Biomedical Engineering and Technology, Chinese Academy of Sciences, Suzhou, China; ^3^ Mechanical Department, Faculty of Engineering at Shoubra, Benha University, Cairo, Egypt; ^4^ Mechatronics and Robotics Department, School of Innovative Design Engineering, Egypt-Japan University of Science and Technology, Alexandria, Egypt

**Keywords:** soft pneumatic muscle, ankle rehabilitation, ankle mobility, rotational movement, geometrical parameters, finite element simulation, deep-learning position estimation models, and silicone-based materials

## Abstract

**Introduction:** A soft pneumatic muscle was developed to replicate intricate ankle motions essential for rehabilitation, with a specific focus on rotational movement along the x-axis, crucial for walking. The design incorporated precise geometrical parameters and air pressure regulation to enable controlled expansion and motion.

**Methods:** The muscle’s response was evaluated under pressure conditions ranging from 100-145 kPa. To optimize the muscle design, finite element simulation was employed to analyze its performance in terms of motion range, force generation, and energy efficiency. An experimental platform was created to assess the muscle’s deformation, utilizing advanced techniques such as high-resolution imaging and deep-learning position estimation models for accurate measurements. The fabrication process involved silicone-based materials and 3D-printed molds, enabling precise control and customization of muscle expansion and contraction.

**Results:** The experimental results demonstrated that, under a pressure of 145 kPa, the y-axis deformation (y-def) reached 165 mm, while the x-axis and z-axis deformations were significantly smaller at 0.056 mm and 0.0376 mm, respectively, highlighting the predominant elongation in the y-axis resulting from pressure actuation. The soft muscle model featured a single chamber constructed from silicone rubber, and the visually illustrated and detailed geometrical parameters played a critical role in its functionality, allowing systematic manipulation to meet specific application requirements.

**Discussion:** The simulation and experimental results provided compelling evidence of the soft muscle design’s adaptability, controllability, and effectiveness, thus establishing a solid foundation for further advancements in ankle rehabilitation and soft robotics. Incorporating this soft muscle into rehabilitation protocols holds significant promise for enhancing ankle mobility and overall ambulatory function, offering new opportunities to tailor rehabilitation interventions and improve motor function restoration.

## 1 Introduction

Assistive robots have long been introduced into the field of rehabilitation. Despite the contentious progression of inflexible robots, these machines still suffer from numerous limitations that prove challenging to overcome. These limitations primarily stem from the materials utilized in their construction, which possess a substantial Young’s modulus, and the inherent constraints imposed by their structures. Simultaneously, rehabilitation exercises often require high flexibility and freedom of movement. Traditional rehabilitation exoskeletons, predominantly composed of rigid components such as linkages and hinges, tend to increase the weight borne by patients, thus inducing discomfort. Moreover, the attainment of multi-degree-of-freedom motion requires additional drive components and intricate structures (Sanjuan et al., 2020). Consequently, the resulting structure becomes excessively intricate, less pliable, and uncomfortable for the patient. Although rigid rehabilitation exoskeletons have already incorporated sophisticated sensor systems to enable perception and interaction with the external environment, their notable hardness, high density, and inability to deform in response to external forces continue to pose substantial safety risks. These machines rely heavily on their structure for all movements, exhibiting poor adaptability (Pérez Vidal et al., 2021).

In contrast to rigid rehabilitation robots, which have inherent limitations, soft robots offer distinctive advantages in terms of safety and adaptability. The abundance of degrees of freedom in soft robots effectively compensates for many of the limitations encountered with their rigid counterparts, allowing greater range of motion and versatility (Su et al., 2022). Soft rehabilitation robots possess the ability to bend, twist, and stretch significantly, allowing them to adapt their shape to accommodate various geometric parameters or encountered obstacles. This inherent flexibility proves particularly advantageous in confined spaces, where precise motions and intricate object grasping are required (Liu et al., 2023).

Composed of flexible materials that mimic biological properties, soft rehabilitation robots offer high adaptability, flexibility, and safety, specifically regarding human-computer interaction and pneumatic-driven actuators (Peng and Huang, 2019). This design allows them to more accurately emulate physical movements and uncover underlying biological principles, making them well-suited for clinical and outdoor rehabilitation settings. By effectively addressing the limitations of traditional rigid rehabilitation robots, soft robots have found significant utility in specific applications within the rehabilitation field. Notably, actuator-based bionic soft robots have emerged as a prominent and extensively researched topic (Elsamanty et al., 2012; Elsamanty et al., 2013), finding widespread utilization in the medical device industry (Pan et al., 2022). Consequently, the development and utilization of soft robots represent a substantial advancement in rehabilitation, offering enhanced safety, adaptability, and functionality compared to their rigid counterparts.

Over the years, artificial muscles have garnered increasing interest, with their initial prototypes dating back several years (Craddock et al., 2022). One notable pioneering invention in this domain is the braided pneumatic actuator developed by McKibben, primarily intended for orthotic applications in patients with spinal muscular atrophy. This actuator comprises an internal rubber tube and an external braided sleeve. Upon inflation, the internal rubber tube expands in volume while the length of the braided sleeve remains constant, resulting in radial expansion and axial contraction of the actuator (Yahara et al., 2019; Youssef et al., 2022). Although this actuator represented a significant breakthrough at the time, it was not without its limitations. Notably, hysteresis stemming from friction between the inner and outer components and a slower contraction rate was notable drawbacks. Nonetheless, these early advancements laid the foundation for ongoing research and development in artificial muscles (Kalita et al., 2022). The ongoing interest in artificial muscles has driven efforts to address the limitations encountered in the initial prototypes, aiming to enhance performance and broaden potential applications. Researchers have explored various mechanisms and materials beyond pneumatic actuators, including electroactive polymers, shape-memory alloys, and liquid crystal elastomers. These alternative approaches offer advantages regarding response time, force generation, and controllability, overcoming the drawbacks associated with traditional pneumatic actuators.

A two-dimensional design of elastic muscles was proposed (Park et al., 2014). In its passive state, the artificial muscle assumes a flat two-dimensional configuration, thereby enhancing the compactness of the artificial muscle mechanism and significantly simplifying the manufacturing process. A universal design method for double helix McKibben muscles was proposed, expanding the application of the double helix McKibben muscle across the entire design space (Bishop-Moser and Kota, 2015). However, these artificial muscles, which are actuated pneumatically using soft materials, possess a notable drawback: they are susceptible to damage from excessive internal gas pressure or contact with sharp external objects. Self-healing soft material (SH) was introduced and successfully applied to manufacturing artificial muscles (Terryn et al., 2017; Elsamanty et al., 2021) to address this limitation. The self-healing property enables the damaged area to heal through mild heating for a short time, thereby greatly enhancing the anti-damage capability of artificial muscles. Furthermore, a pneumatic joint based on an origami structure was proposed to improve soft actuators’ rotational stiffness and range (Shoushtari et al., 2019). This innovative approach contributes to the advancement of soft actuators in terms of their performance characteristics.

A bubble artificial muscle (BAM) was proposed, which differs from the radial pleated muscle. This artificial muscle restricts its expansion by placing metal rings on plastic tubes, forming circumferential pleats, and dividing them into several units. This design achieves a similar contraction rate of 45%–50% (Saleh et al., 2020; Diteesawat et al., 2021). The RoboThespian, a PAM-actuated humanoid robot, was developed by Engineered Arts Limited from Cornwall for education, communication, interaction, and entertainment. A soft pneumatic gripper fabricated from TPU material using 3D-printed FDM technology was proposed (Saleh et al., 2020). This gripper can accommodate fruits of various shapes and sizes without compromising bioimpedance functionality by employing an appropriate gripping force (Saleh et al., 2020b). The researchers investigated the effect of changing the air pillow inclination angle on the work envelope of the soft muscle and the reaction force applied to the tip of the soft muscle. This was achieved by applying positive and vacuum pressure on the inner surfaces of the soft muscle (Saleh et al., 2020). The concept of a fiber-reinforced bending soft muscle was introduced to estimate the value of the material constant of 3D-printed soft muscle. The researchers developed a model and performed an analysis using ABAQUS software (FEM). Subsequently, they conducted experimental verification by prototyping the model and subjecting it to stimulation at various frequencies. The experimental results demonstrated that the proposed method for obtaining the material constant could accurately predict the behavior of prototypes in real experiments, encompassing a range of pressure or deflection (Gharavi et al., 2022).

In the realm of rehabilitation technology, significant advancements have been made in the development of exoskeleton-assisted systems, particularly in the field of upper limb rehabilitation (Masengo et al., 2020; GUO et al., 2022). One noteworthy innovation is the low-cost and customizable 3D-printed hand exoskeleton, which offers a versatile solution for individuals undergoing hand injury rehabilitation. This groundbreaking device facilitates the restoration of dexterity and mobility and demonstrates the potential to adapt to individual needs (Rudd et al., 2019). Moreover, a lightweight and user-friendly soft robotic exoskeleton system has shown remarkable effectiveness in enhancing hand dexterity and providing valuable assistance during rehabilitation. Integrating soft robotic components in this system contributes to its user-friendly nature and ability to optimize hand movements (Kladovasilakis et al., 2023). The positive outcomes observed in the application of this system further validate its potential as an integral tool for hand rehabilitation.

Furthermore, recent developments have focused on incorporating synchronized fingertip haptic stimulation into exoskeleton-assisted hand rehabilitation systems. These innovative approaches have demonstrated promising results, particularly in enhancing attention levels and user engagement, especially when heavier grasping weights are involved. Including haptic stimulation has proven to be instrumental in improving rehabilitation outcomes and promoting active user participation (Li et al., 2021; Chang et al., 2022). The collective progress in exoskeleton-assisted rehabilitation systems is poised to significantly impact hand rehabilitation outcomes, fostering greater patient engagement and participation. A highly flexible bio-inspired modular soft robotic arm, constructed using fabric TPU, has also emerged as a notable advancement in upper limb robotics (Hernandez-Barraza et al., 2023). This arm offers customizable joints and exhibits distinct bending patterns corresponding to different motions at varying pressures. The design incorporates seamlessly combinable joint and link sections, allowing customization based on specific requirements. The remarkable flexibility, adaptability, and potential for various applications make this bio-inspired arm a promising development in upper limb robotics (Hernandez-Barraza et al., 2023).

A comprehensive approach to wrist rehabilitation utilizes a compact and low-profile soft robotic wrist brace constructed from ethylene-vinyl acetate material. Integrating eight soft origami-patterned actuators onto a commercially available brace significantly enhances its functionality. Furthermore, the adoption of blow molding techniques enables cost-effective mass production of these actuators, ensuring the scalability and reproducibility of the device (Liu et al., 2021). A series of extensive experimental evaluations have been conducted to gain deeper insights into the capabilities and limitations of the wrist brace. These evaluations provide valuable empirical evidence supporting the brace’s efficacy as a promising solution for wrist rehabilitation, thereby improving individuals’ quality of life who suffer from wrist-related impairments or injuries (Liu et al., 2021). Additionally, the investigation encompasses the development of a supernumerary robotic limb aimed at mitigating injuries and reducing the joint load in the upper limb for workers. This advanced system incorporates a wearable gravity compensation mechanism, a soft robotic hand, and a custom damping wrist, constituting an effective solution to address work-related musculoskeletal disorders (WMSD) (Ciullo et al., 2021).

The experimental analysis unveils noteworthy enhancements achieved by the robotic limb system compared to traditional hand drilling methods. Particularly significant is the reduction in vibration transmission by an impressive range of 40%–60%, all while maintaining satisfactory time performance. These findings underscore the potential of the developed system to enhance worker safety, minimize the incidence of injuries, and optimize workplace ergonomics and worker wellbeing (Ciullo et al., 2021). The comprehensive approach to wrist rehabilitation demonstrated in this study involves the utilization of a compact and low-profile soft robotic wrist brace. Furthermore, developing a supernumerary robotic limb represents a significant advancement in mitigating injuries and alleviating joint load in the upper limb for workers. The empirical findings highlight the potential of this system to improve worker safety, minimize work-related musculoskeletal disorders, and optimize workplace ergonomics, thus contributing to the wellbeing and productivity of individuals in occupational settings (Rudd et al., 2019; Ciullo et al., 2021; Liu et al., 2021; Kladovasilakis et al., 2023).

An investigation introduces a wearable system that integrates various assistive technologies for individuals with upper-limb impairments (Guo et al., 2023; Orban et al., 2022). The system combines sensory components, haptic feedback mechanisms, orthotic devices, and robotics to facilitate forearm lifting and enhance grasping capabilities. One notable feature of this wearable system is incorporating a robotic supernumerary finger, further enhancing users’ functional abilities. Through real-world scenarios, the effectiveness of the developed wearable system has been demonstrated, underscoring its practical value and potential to significantly improve the quality of life and promote independence among individuals with upper-limb impairments (Salvietti et al., 2021). Another study presents a similar wearable system that integrates assistive technologies for individuals with upper-limb impairments. The system, similar in design and functionality, offers enhanced grasping and forearm lifting capabilities by integrating sensory, haptic, orthotic, and robotic components. The positive outcomes observed in real-world scenarios further emphasize this wearable system’s practical value and potential impact in enhancing individuals’ independence and overall quality of life who are affected by upper-limb impairments (Salvietti et al., 2021).

A novel 4-degree-of-freedom (DOF) lower limb rehabilitation robot has been introduced, offering flexion/extension (F/E) training for three limb joints and adduction/abduction (A/A) training for the hip joint (Wang et al., 2020). This innovative robotic system allows for direct wheelchair training, eliminating the need for frequent patient handling and ensuring safety through comprehensive joint motion analysis and validated trajectory planning methods (Alphonse et al., 2019; Elkholy et al., 2020). Integrating these advancements in lower limb rehabilitation robotics opens new possibilities for comprehensive training programs and improved rehabilitation outcomes. Additionally, an analysis and design optimization study focuses on the actuation system of a soft module lower limb exoskeleton, resulting in substantial improvements in energy efficiency and overall performance (Ortiz et al., 2018). By implementing optimization techniques based on user needs and gait data, significant reductions in energy requirements, ranging from 20% to 65%, have been achieved across specific joints. Ongoing efforts within the XoSoft EU project aim to refine further and validate the optimized mechanism, thereby contributing to the continued enhancement of energy efficiency and functional effectiveness in soft module lower limb exoskeletons.

A soft-pneumatic actuator-driven exoskeleton designed specifically for hip flexion rehabilitation is presented in this study. Comprehensive testing and evaluation have confirmed the effectiveness of the exoskeleton in assisting hip flexion movements, generating substantial torque, and reducing muscle effort. These findings underscore the exoskeleton’s potential as a valuable tool for facilitating efficient and effective hip flexion rehabilitation and alleviating muscle burden (Miller-Jackson et al., 2022). In another investigation focusing on human postural adjustments on compliant surfaces, it has been observed that repetitive movements play a crucial role in enabling individuals to acquire knowledge of stiffness profiles. Notably, differences in learning stages have been identified for various stiffness profiles. Despite these variations, position estimation remains consistent across different stiffness profiles, while force estimation accuracy varies depending on the specific profile (Takahashi et al., 2022).

Moreover, a bio-inspired controller that leverages motor primitives has been proposed for a lower limb exoskeleton. This controller effectively compensates for torque deficiencies and accommodates variations in gait characteristics, thus enhancing motor performance and synchronization between the human and exoskeleton system. The effectiveness of the motor primitive-based controller has been demonstrated in addressing motor deficiencies during lower limb movements (Nunes et al., 2020). A novel method for feature extraction and classification of lower limb motion using sEMG signals has also been introduced. This method integrates WPT, PCA, SUKF, and NN to enhance accuracy and reliability, resulting in an impressive average accuracy of 93.7%. The advancements made through this method contribute significantly to lower limb motion analysis and the development of more accurate motion classification systems (Shi et al., 2020).

Focusing on the limitations of rigid rehabilitation robots and the advantages of soft robots in rehabilitation. Despite integrating sensor systems, rigid rehabilitation exoskeletons still pose safety risks due to their hardness, high density, and inability to deform in response to external forces. In contrast, soft rehabilitation robots, with their flexibility and adaptability, have found utility in specific rehabilitation applications. However, existing soft actuators, such as artificial muscles actuated pneumatically, are susceptible to damage and limitations in performance. Therefore, there is a need for improved soft actuators that overcome these drawbacks. The paper aims to explore advancements in wearable systems and lower limb rehabilitation robots, specifically focusing on ankle rehabilitation. The paper aims to propose a compact and low-profile soft robotic ankle brace and investigate the development of a supernumerary robotic limb to mitigate injuries and reduce the lower limb’s joint load. The goal is to enhance individuals’ functional abilities and improve rehabilitation outcomes through innovative technologies and approaches tailored to ankle rehabilitation.

## 2 Soft muscle application and design

**FIGURE 1:**
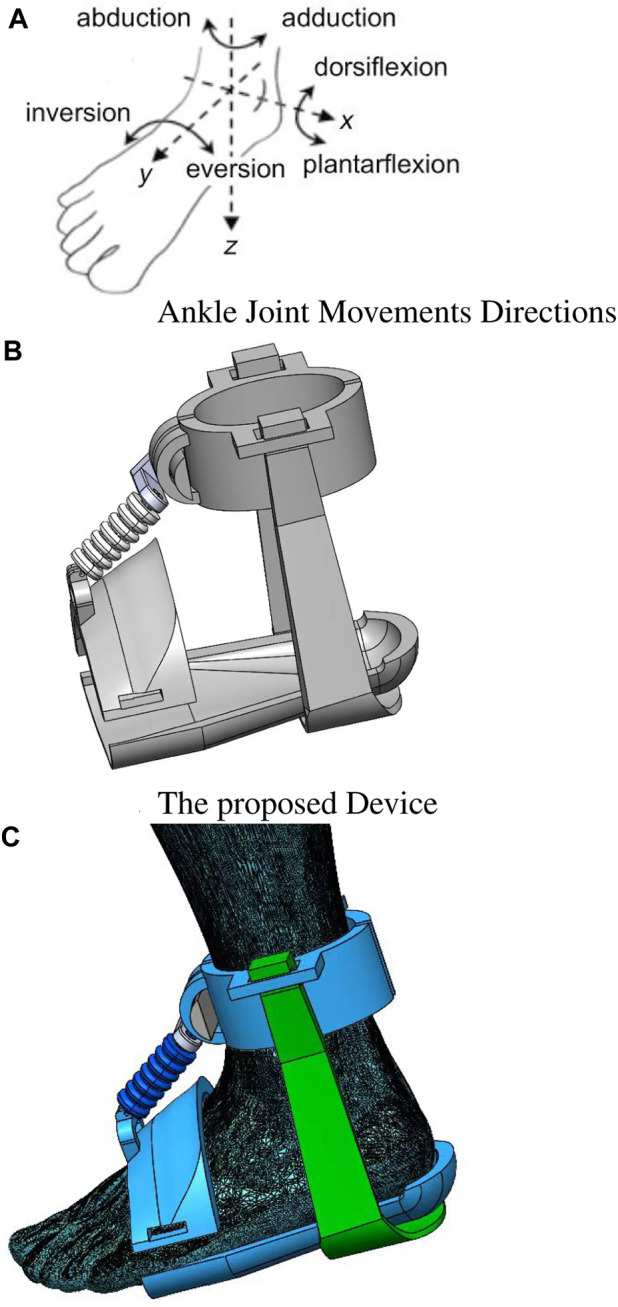
The real application for our designed muscle showcasing the following components. **(A)** Illustration highlighting the different directions of movement at the ankle joint, indicating flexion, extension, inversion, and eversion. **(B)** Schematic representation of the designed device specifically tailored for ankle muscle control and movement. **(C)** Installation of the ankle muscle the same as **(A,B)**. The figure illustrates the proper alignment and attachment of the muscle, ensuring optimal functionality and range of motion.

In the human leg, four muscles are crucial in facilitating ankle rotation movement: Peroneus brevis, Peroneus longus, Extensor digitorum longus, and Tibialis anterior (Zeng et al., 2020; Shah et al., 2022). However, peripheral nerve damage, stroke, hemiplegia, tumor muscle, and bone damage can adversely affect the patient’s ability to walk effectively. While medication and surgical interventions may address the underlying issues, rehabilitation exercise therapy remains essential in restoring walking ability and muscle strength. The ankle joint exhibits three primary motions: dorsiflexion, plantarflexion, and inversion/eversion, as depicted in Figure 1A. The proposed soft muscle is specifically designed to actuate the rotational movement along the *x*-axis, as this movement is pivotal in supporting the patient’s walking ability. Incorporating the soft muscle into the rehabilitation process is expected to assist patients in regaining ankle mobility and enhancing their overall ambulatory function.

**FIGURE 2:**
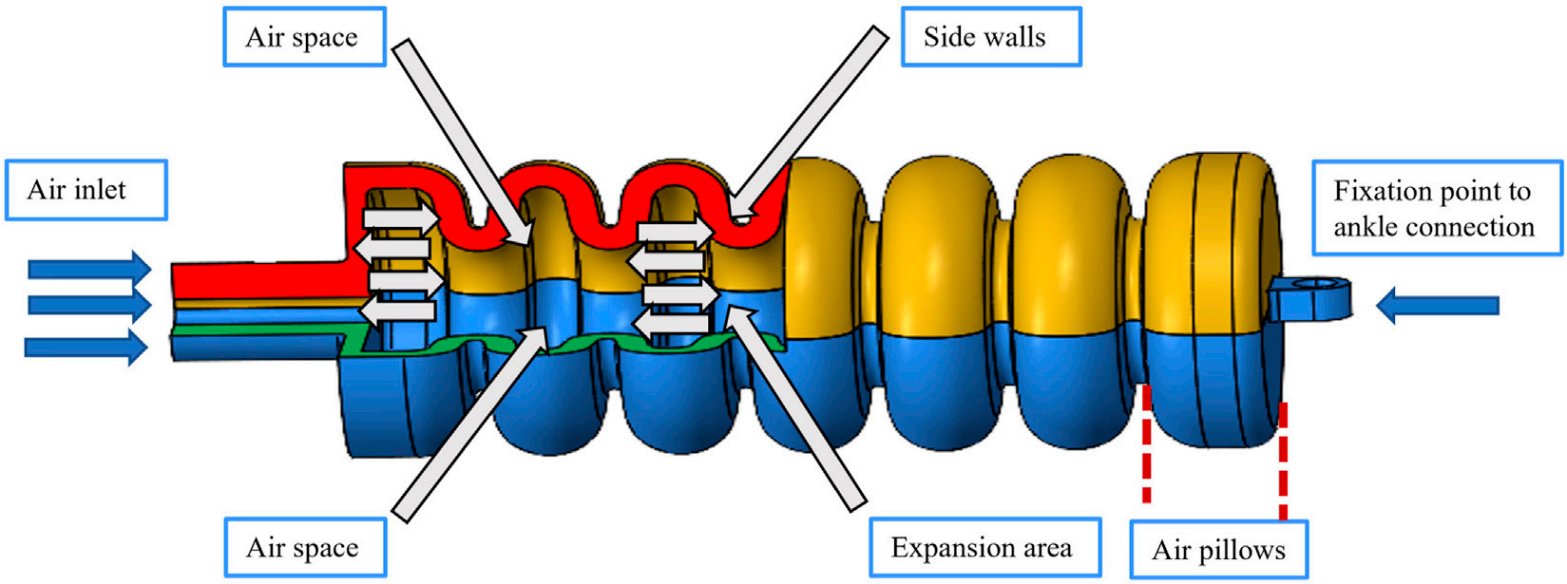
The soft muscle internal details represent the internal structure of the soft muscle and the air tubes, the affected walls with the pressure.

In this study, a novel soft muscle design is proposed to replicate the complex movements of the ankle joint. As illustrated in Figure 1, the soft muscle design incorporates various geometrical parameters, such as the air below thickness and the distance between the air below. These parameters are carefully chosen to ensure accurate mimicry of the ankle’s twisting movement, as depicted in Figure 1B. The design of the soft muscle is based on the Fast pneu-nets Grid Structure design. This structure allows for expansion through air pillows positioned between the sidewalls. By expanding in a controlled manner, the muscle increases the length necessary for facilitating the rotation of the ankle around its *X*-axis, as exemplified in Figure 1C. The soft-driven joint primarily consists of multiple air pillows. When subjected to external air pressure, the inner wall of the air pillows undergoes expansion and deformation, increasing the length of the drive layers. This mechanism enables the soft muscle to generate the desired range of motion and accurately replicate the complex movements of the ankle joint. By employing this innovative soft muscle design as shown in Figure 2, it is anticipated that improved functionality and performance can be achieved in applications related to ankle joint rehabilitation and assistive devices. The design provides a promising avenue for enhancing the effectiveness of rehabilitation therapies and contributing to the overall wellbeing and mobility of individuals with ankle impairments.

**FIGURE 3:**
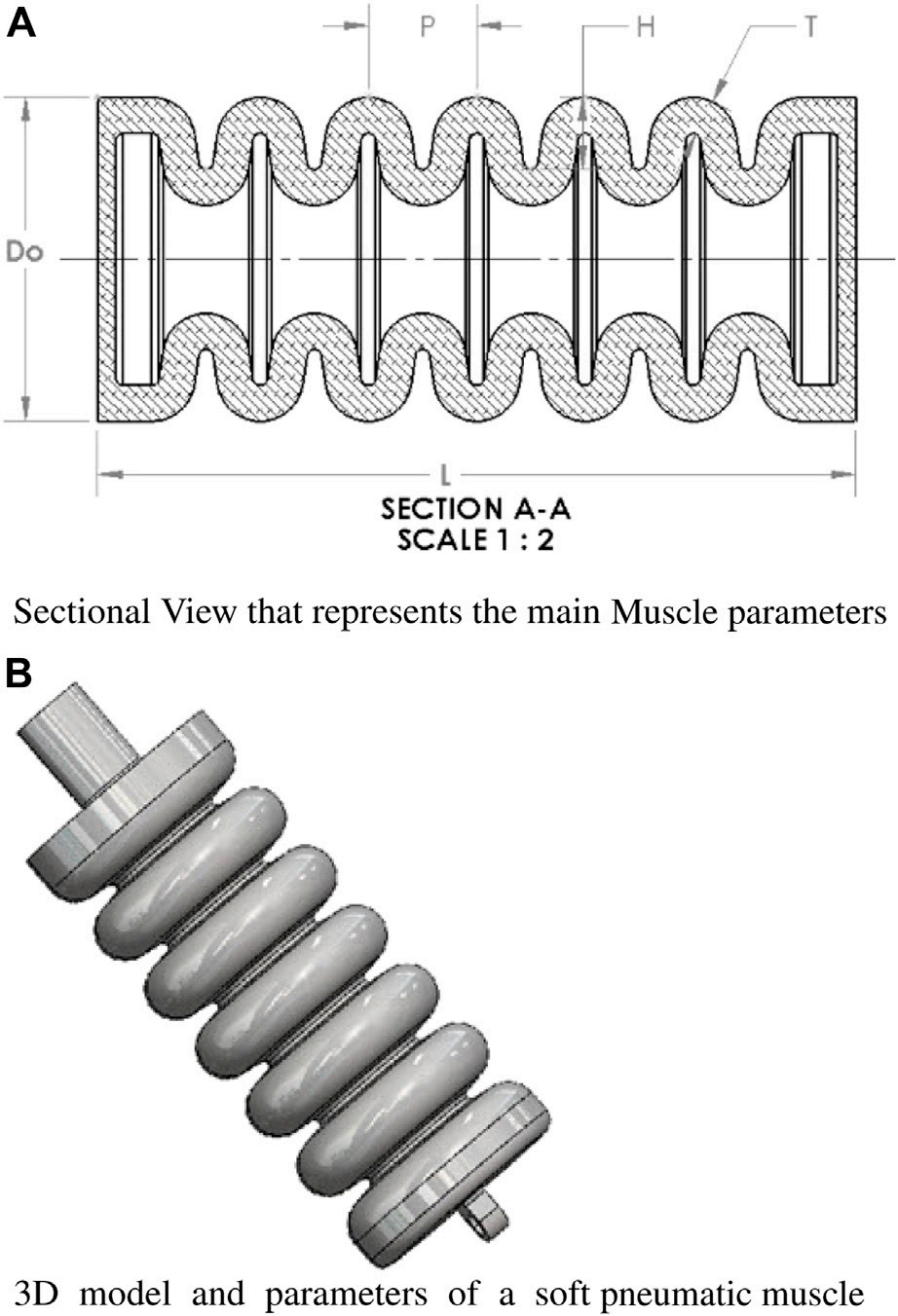
Visualization and Parameter Analysis of a Soft Pneumatic Muscle. The figure provides a comprehensive depiction of the soft pneumatic muscle, emphasizing its design features and significant parameters. **(A)** a sectional view is presented, elucidating the main muscle parameters. **(B)** Showcases the detailed 3D model and associated parameters of the soft pneumatic muscle.

The present study employs a systematic approach for selecting geometrical parameters to achieve optimal performance of the soft pneumatic muscles. A rigorous benchmarking process was undertaken, considering various factors such as motion range, force generation, and energy efficiency. The resulting model showcases promising motions that hold potential applications in post-stroke patient rehabilitation and physical therapy settings. To gain a comprehensive understanding of the soft muscle’s behavior, an in-depth analysis was carried out. This analysis evaluated the muscle’s motion under different conditions, specifically on predetermined air pressure values. By examining the muscle’s response across various pressures, valuable insights were obtained regarding its performance characteristics. The selected geometrical parameters, critical to the soft muscle’s functionality, are visually illustrated in Figure 3 and detailed in Table 1. This comprehensive representation provides a clear overview of the key design attributes and facilitates a deeper understanding of the muscle’s underlying mechanics. Researchers and practitioners can systematically manipulate these parameters to tailor the muscle’s performance to meet specific application requirements. The meticulous selection of geometrical parameters, coupled with the thorough analysis conducted, enhances our understanding of the soft pneumatic muscle’s capabilities. This knowledge serves as a foundation for further advancements in rehabilitation and physical therapy, offering opportunities to improve the effectiveness of interventions for post-stroke patients and individuals seeking to regain motor function.

**TABLE 1:** Geometrical parameters of soft pneumatic muscles (SPMs).

Parameter	Value
Length (L)	105
Outer Diameter (*D*_*o*_)	45
Height (H)	5
Pitch (P)	15
Thickness (T)	3.5

The design represents a single chamber but also contains a multi-air pillow is better to mention it as a multi-air pillow constructed from a flexible elastomeric material, specifically silicone rubber. Instead of a traditional reinforcement layer, chamber rings provide structural integrity. The controlled expansion of the soft muscle is achieved through the pressurization of these chambers with air. Key parameters governing the behavior of the soft muscle include the chamber length (L), outer diameter (*D*_*o*_), chamber height (H), pitch (P), and chamber thickness (T), as outlined in Table 1. The chamber thickness is 3.5 mm, utilizing a silicone rubber material (BT903) known for its expandable properties. The appropriate material selection is crucial as it influences the muscle’s ability to undergo extension and contraction.

Careful consideration is given to the air pressure and flow rate to facilitate the desired muscle performance. Pressurized air is utilized to induce extension in the soft muscle. Optimal material selection, size, shape, air pressure control, and flow rate are critical to achieving the desired muscle behavior. In this study, typically applied pressures within the soft muscle fall within the 105–140 kPa range. By comprehensively addressing these factors, the design and implementation of the soft muscle model are optimized to ensure reliable and controlled muscle expansion. This knowledge paves the way for further advancements in developing soft pneumatic muscles, enabling their integration into various applications, such as rehabilitation robotics, prosthetics, and assistive devices.

## 3 Enhancing soft muscle design

The prediction and enhancement of soft muscle designs necessitate a comprehensive understanding of their mechanical performance. This understanding can be attained through empirical tests and advanced finite element simulation programs, such as Ansys software, widely employed in Finite Element analysis (Ge et al., 2018; Wang et al., 2018). The soft muscle is constructed using a silicone rubber material (BT903), renowned for its favorable characteristics. Its low viscosity and high fluidity facilitates convenient shaping, while its exceptional elasticity, high tensile strength, and resistance to aging contribute to the durability and longevity of the soft muscle. For further details, please refer to Table 2.

**TABLE 2:** BT903 Silicone performance parameters.

Parameter	Numerical values
Hardness (A)	39
Tensile Strength (MPa)	2.88 MPa
Tear Strength (kN/m^2^)	23 kN/m^2^
Mixing Ratio (A: B) grams	100:2
Curing Time at Room Temperature	5–6 (hr)

**FIGURE 4:**
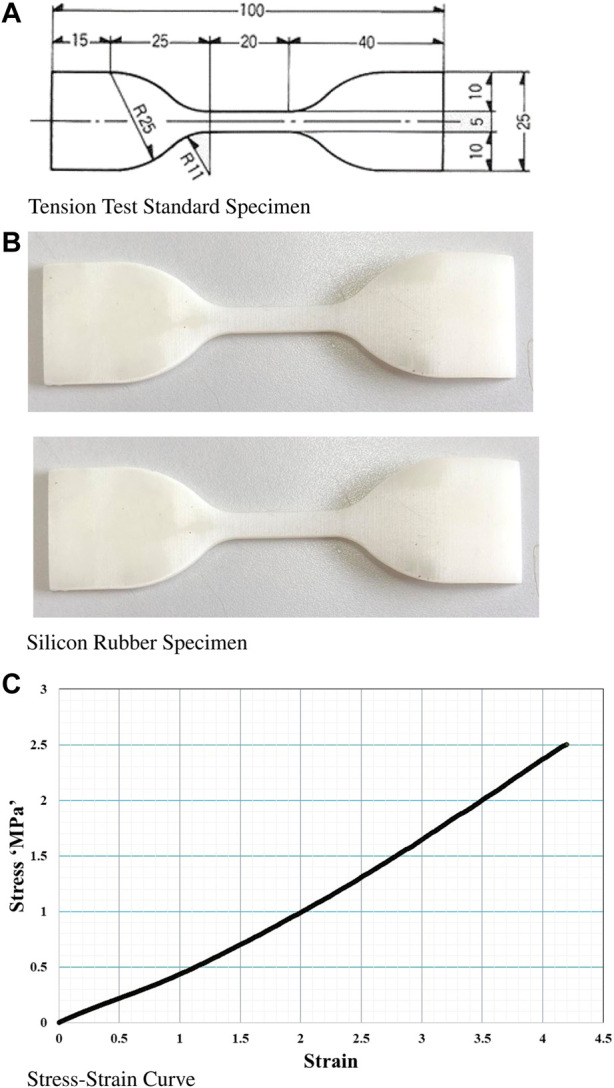
**(A)** Geometrical dimensions and manufacturing of ASTM D412 specimens of Silicone rubber material. **(B)** Fabricated ASTM D412 specimen of Silicone rubber after curing. **(C)** Experimental results: Stress-strain curve for the tested Silicone rubber material.

In the context of finite element simulation, the meticulous selection of an appropriate material holds paramount importance as it directly influences the accuracy and reliability of the model. Thus, to ensure precise predictions, it is imperative to integrate the mechanical properties of the chosen material, including material constants obtained from mechanical testing procedures such as the mechanical tension test as shown in Figure 4. This test follows the specifications outlined in the ASTM D412 standard and enables the acquisition of the stress-strain curve [44, 45]. It is worth noting that inherent variability in the properties obtained from these tests may introduce certain degrees of variation in the simulation results (Kalisky et al., 2017; Steck et al., 2019).

Rigorous and reliable simulations are of utmost importance to guarantee accuracy and optimize the simulation process. Among the key factors in establishing a robust simulation model are the material constants *C*_10_, *C*_20_, and *C*_30_. The tensile test is the standard method for determining material properties in soft muscle design. This test entails the fabrication of a specially designed specimen that adheres to the specifications outlined in the ASTM D412 standard. Subsequently, the fabricated specimen undergoes meticulous testing using specialized instruments (Renaud et al., 2009). A comprehensive curve-fitting analysis was conducted to identify the most suitable hyperelastic model. The Yeoh 3rd-order hyperelastic material model was selected in this study due to its compatibility in simulating materials with large strain differences ranging from 0% to 500% (Saleh et al., 2020). Previous research has demonstrated the superior performance of the Yeoh model compared to other hyperelastic material models (Masengo et al., 2020; GUO et al., 2022). The general formula for hyperelastic material models is represented by Eq. 1 (Rudd et al., 2019; Kladovasilakis et al., 2023):

$$W=\overset{N}{\underset{n=1}{\sum }}\left(\frac{dk}{C_{in}}\right){\left({\bar{I}}_{1}-3\right)}^{n}$$ (1)

Here, *W* denotes the strain energy, *N* represents the number of terms in the series, ${\bar{I}}_{1}$ is the first derivative of the strain invariant, *J* is the determinant of the elastic deformation gradient $\left({\bar{I}}_{1}=J^{-2/3}I_{1}\right)$ , and *C*_*i*0_ and *dk* are material constants. However, in the Yeoh model, the incompressibility constraint is neglected. The value of *C*_*i*0_ is crucial for conducting the simulation. To streamline the simulation process, it opted to use the first order of the Yeoh model (*N* = 3). Where the only material constant required by the simulation software is *C*_30_.

For the purposes of simulating the model in this study, finite element analysis was performed using ANSYS. The prototype was subjected to simulations ranging from a pressure of 105 KPa to 145 KPa. Then, the material parameters derived from the curve fitting process were subsequently integrated into the FEA software (ANSYS) to establish the finite element analysis. Detailed information regarding the specific material parameters of the hyperelastic model can be found in Table 3, serving as a valuable point of reference for further analysis.

**TABLE 3:** The parameters of Yeoh third order model of Silicon rubber material.

Description	Value
Silicon Rubber Density	1080 kg/m^3^
*C*_10_ (MPa)	0.09183 MPa
*C*_20_ (MPa)	0.0070104 MPa
*C*_30_ (MPa)	−0.00011671 MPa

## 4 FEA results discussions

This rigorous simulation study systematically evaluated the mechanical response and performance of a soft pneumatic muscle for ankle rehabilitation under various loading conditions. The primary objective of this comprehensive analysis was to determine the maximum deformation along the x, y, and *z*-axes and quantitatively assess the distribution of stress within the structure of the soft muscle. To achieve this objective, the muscle underwent a meticulous examination with a wide range of pressures applied, ranging from 105 to 145 kPa in increments of 5 kPa. The pressure application was thoughtfully implemented, selectively targeting the inner surfaces of the muscle while ensuring that the outer surfaces were maintained at a constant atmospheric pressure.

**FIGURE 5:**
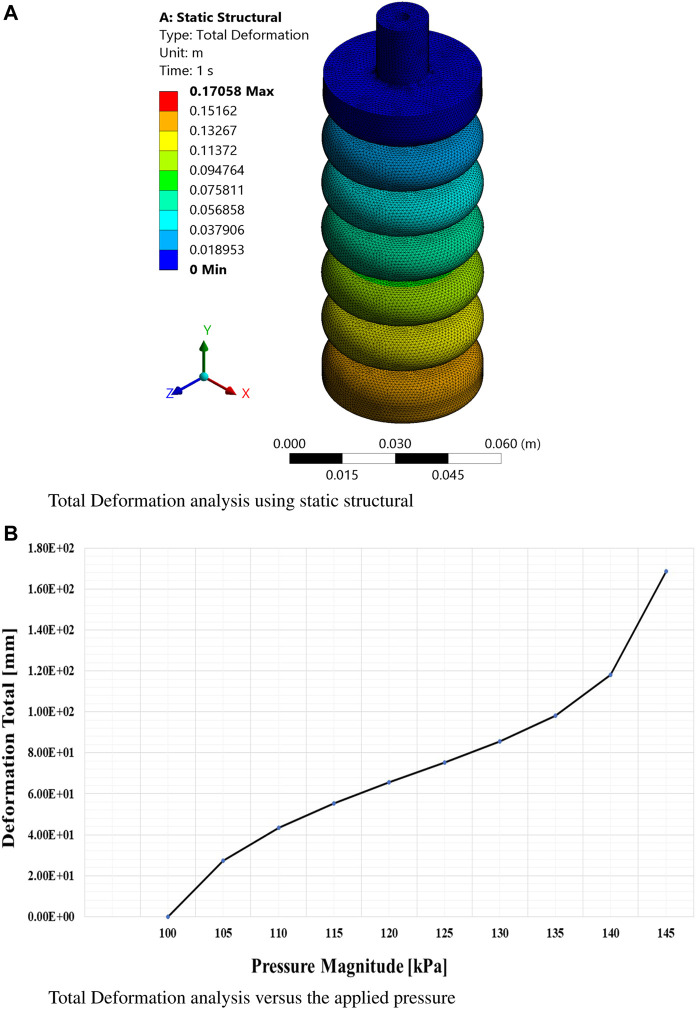
Finite element analysis results at maximum pressure based on the third-order Yeoh hyperelastic model. **(A)** Total deformation analysis using static structural simulations. **(B)** Total deformation as a function of applied pressure.

**FIGURE 6:**
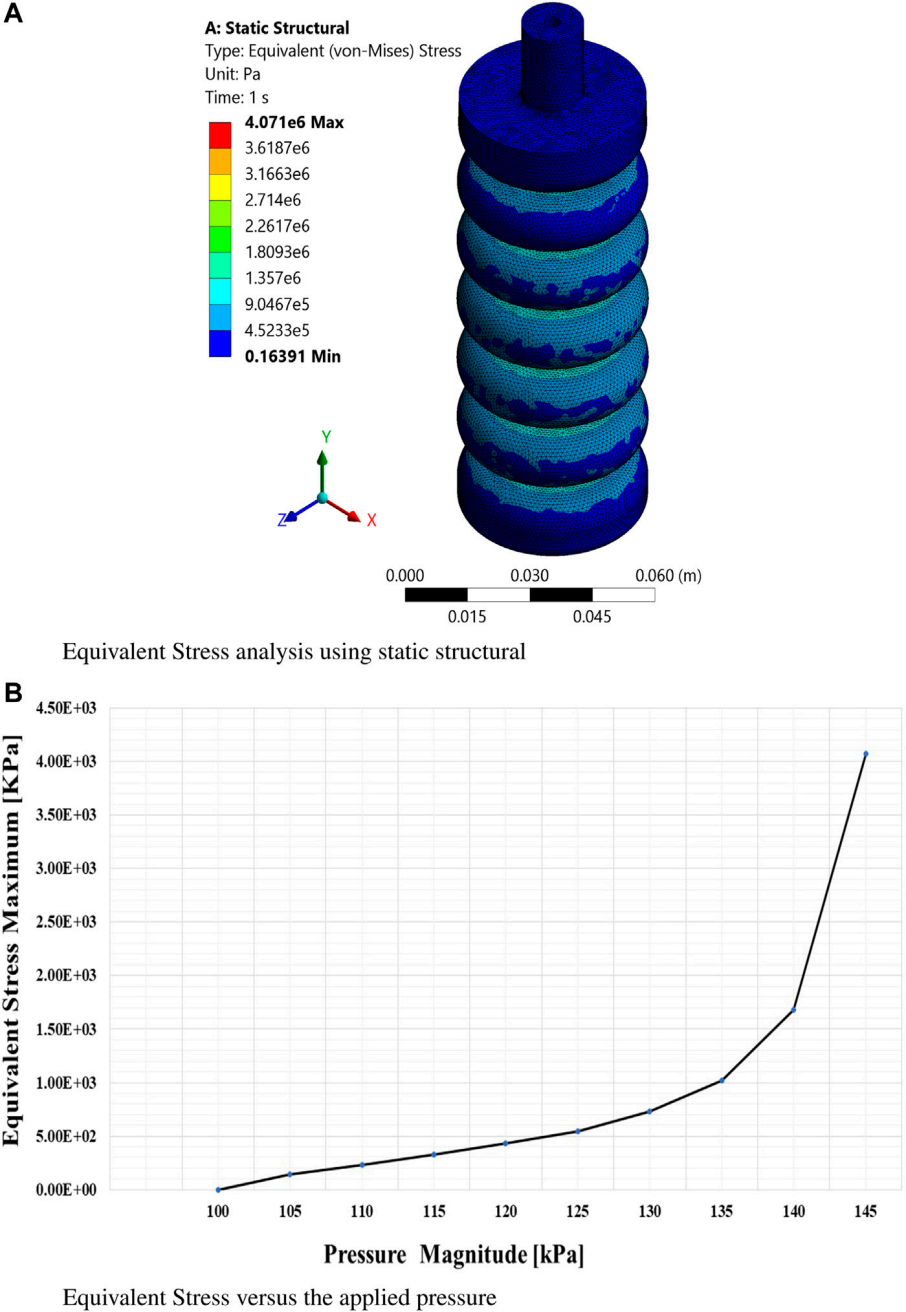
Finite element analysis of equivalent stress. **(A)** Static structural simulation results for equivalent stress at maximum pressure based on the Yeoh hyperelastic model. **(B)** Equivalent stress as a function of applied pressure.

**FIGURE 7:**
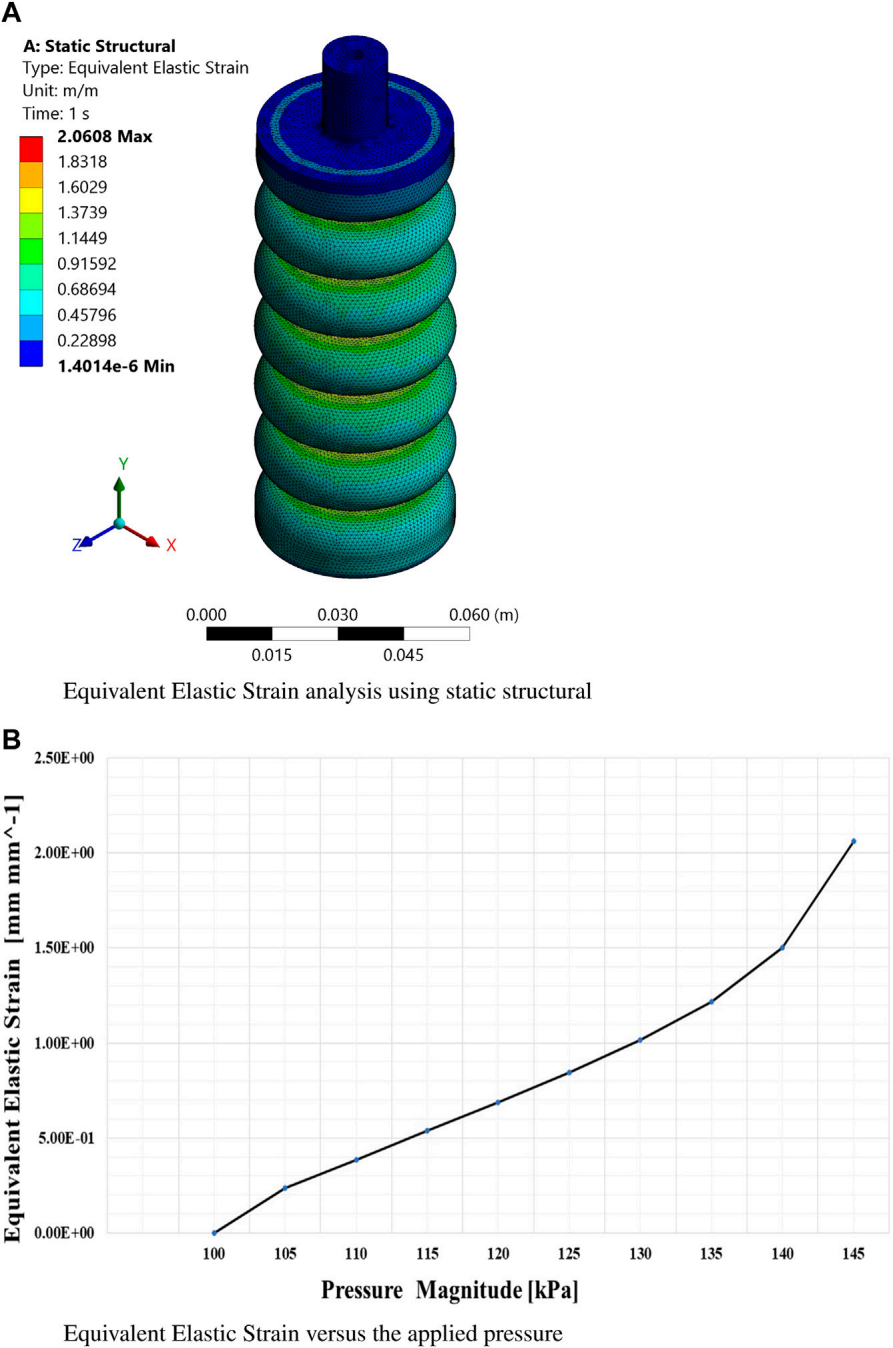
Finite element analysis of equivalent elastic strain. **(A)** Static structural simulation results for equivalent elastic strain at maximum pressure. **(B)** Equivalent elastic strain as a function of applied pressure.

The findings obtained from this extensive simulation analysis, as visually presented in Figure 5, Figure 6, an Figure 7 have significantly advanced our understanding of the intricate mechanical behavior of the soft ankle muscle. These results are of utmost importance as they lay the foundation for the further development and optimization of soft robotics specifically tailored for ankle rehabilitation applications. By systematically investigating the effects of varying input pressures, this study aimed to gain a deep understanding of the characteristics of soft pneumatic muscle deformation and the characteristics of the stress response.

From this meticulous analysis, several notable findings have emerged, providing valuable insights into the performance of the soft muscle with respect to deformation and stress. Remarkably, at a pressure of 145 kPa, the deformation along the *Y*-axis (Y-def) exhibited a substantial measurement of 165 mm, clearly indicating a significant elongation primarily occurring in the *Y*-direction.

This outcome underscores the robustness and efficacy of the soft muscle design, particularly in facilitating ankle flexion and extension. On the contrary, the deformations along the *x* and *z*-axes were negligibly small, measuring 0.056 mm and 0.0376 mm, respectively, indicating minimal elongation along these axes. This observation emphasizes the specificity of the soft muscle response to pressure actuation in the intended direction. In addition, the controllability of the muscle at different pressure values was evident, as the elongation range spanned from 0 mm to 165 mm. This remarkable adaptability and controllability enable precise regulation of the ankle’s rotational angle, thus accommodating individual patient needs and facilitating customized rehabilitation protocols.

**FIGURE 8:**
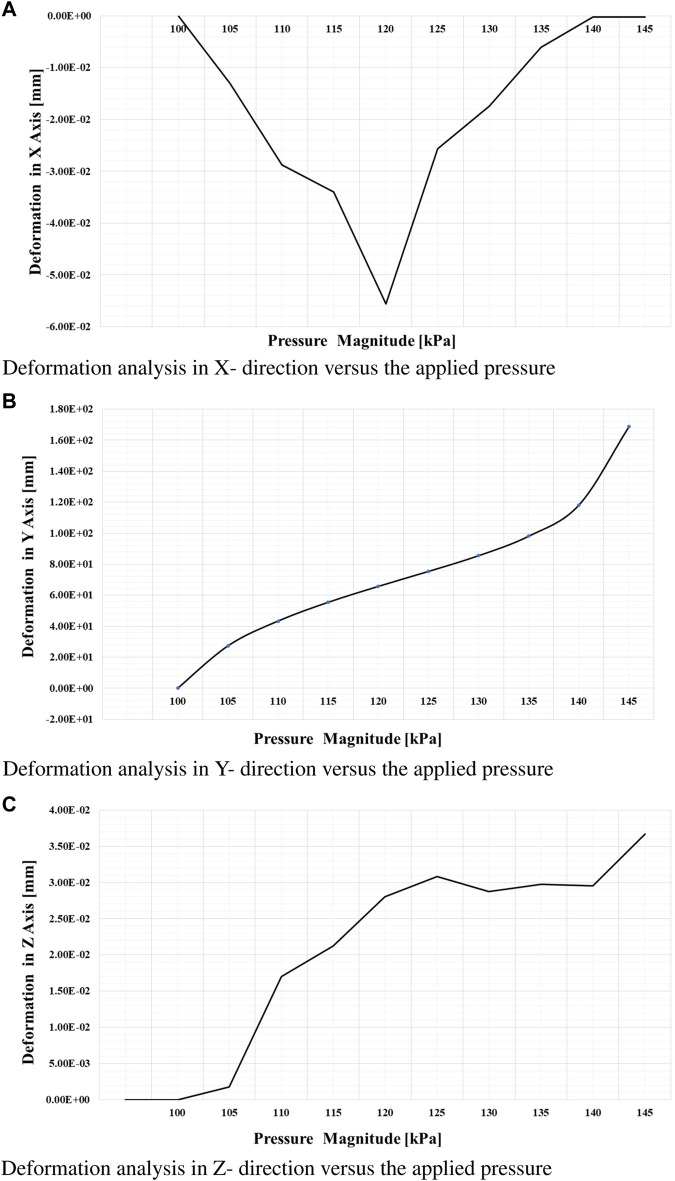
Finite element analysis of deformation in the x, y, and z directions. **(A)** Deformation in the *x*-direction as a function of applied pressure. **(B)** Deformation in the *y*-direction as a function of applied pressure. **(C)** Deformation in the *z*-direction as a function of applied pressure.

The visual representation in Figure 8 confirms and reinforces these findings, effectively illustrating the exceptional adaptability and versatility of the soft muscle design for ankle rehabilitation. In essence, this comprehensive analysis validates the performance of the soft muscle. It is a solid foundation for future research and development efforts in the rapidly evolving field of soft robotics, particularly within medical applications. The invaluable insights gleaned from this meticulous simulation study provide crucial guidance for further exploration and enhancement of soft muscle technology, thus pioneering new possibilities and avenues for improving the efficacy and efficiency of ankle rehabilitation.

## 5 Ankle rehab soft muscle fabrication

The soft muscle fabrication process for ankle rehabilitation is based on advances in soft robotics technology and molding techniques. These methods have attracted significant attention and recognition across various areas, including medical devices, prostheses, and wearable exoskeletons, with various areas attracting this method. Soft muscles have inherent qualities of flexibility and adaptability, making them very promising for rehabilitation applications where patient comfort and safety are paramount. The main purpose of designing soft muscles for ankle rehabilitation is to create devices that faithfully replicate the behavior of natural muscles and tendons, providing the required strength and range of motion for ankle movements such as flexion, extension, inversion, and eversion.

In order to achieve this goal, the correct selection and optimization of the shape and silicone material properties of the mold and silicone material are essential. Because of its elasticity, durability, and biocompatible properties, silicone is an ideal material for prolonged contact with the human body. In addition, silicone enables silicone to be easily molded into custom shapes, allowing soft muscle development to be tailored to an individual’s specific needs and anatomical structure. It also allows for customization. Incorporating air pillows into soft muscle designs precisely controls muscle expansion and contraction. By manipulating the air pressure in the soft muscle, the appropriate level of support or resistance can be imparted during ankle exercises, thus facilitating patient-specific rehabilitation protocols. Additionally, the presence of air pillows increases the comfort of soft muscles, allowing them to adapt to the wearer’s movement accurately. Furthermore, air pillows enhance its overall compliance, allowing it to seamlessly adjust to the wearer’s movements, minimizing any discomfort or inconvenience.

**FIGURE 9:**
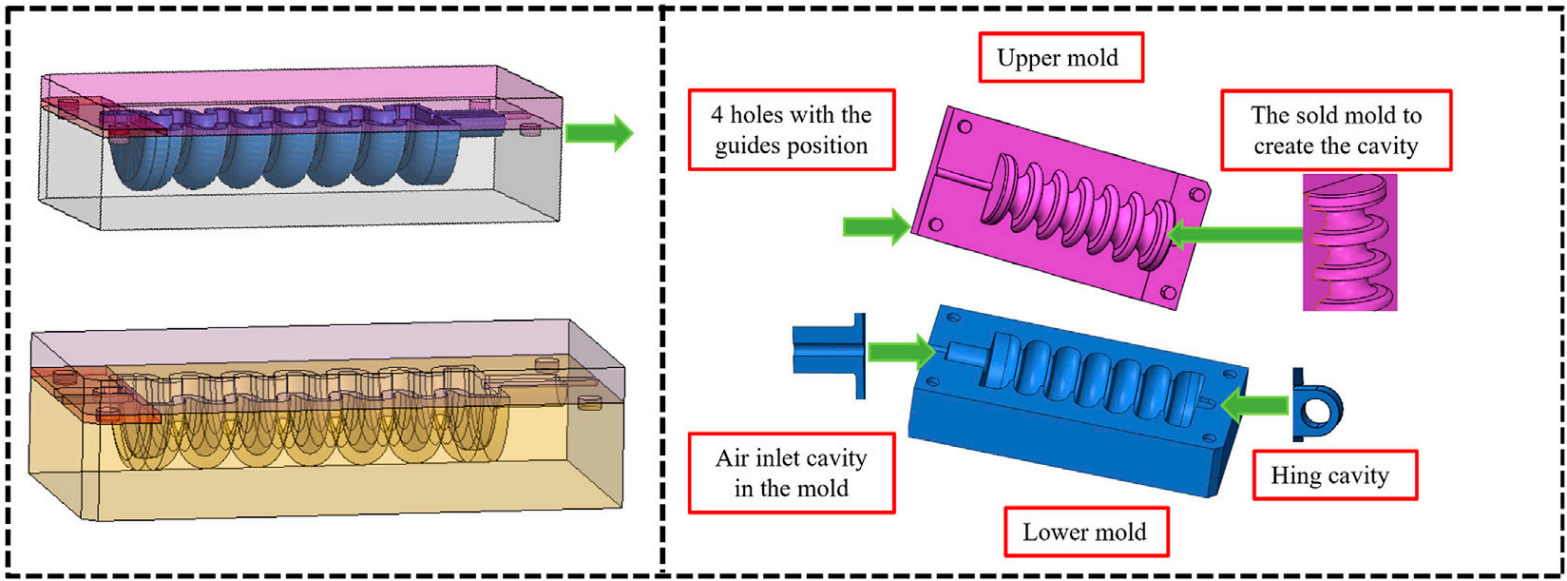
Illustrates the two 3D-printed mold components used in the soft muscle fabrication process for ankle rehabilitation. The lower mold serves as the base for pouring the silicone material, while the upper mold creates the cavity space necessary for forming the air pillows.

**FIGURE 10:**
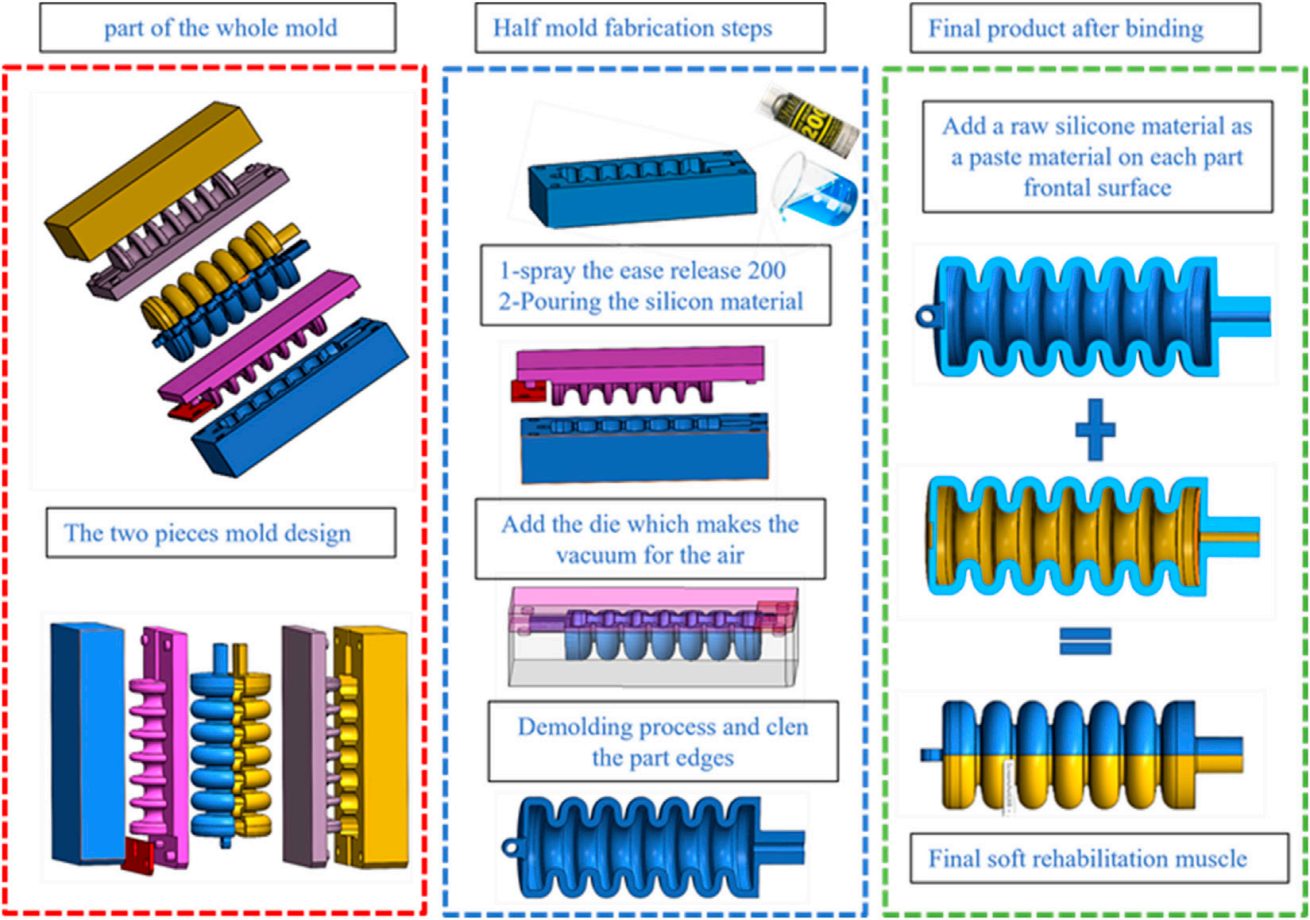
Depicts the detailed fabrication process of the soft muscle for ankle rehabilitation, highlighting the steps involved in mixing and pouring the silicone material and clearly visualizing the mold design components and assembly for creating the final soft muscle product.

The manufacturing process includes the use of silicone-based materials and the creation of a hollow soft muscle model using two distinct 3D printed molds, as shown in Figures 9, 10. Each mold has a lower section of silicone material and an upper section of the cavity space for air pillows that form the cavity. It consists of a lower section where the silicone material is poured and an upper section which is the cavity space for the air pillows. The lower mold has four holes, while the upper mold has four guiding bars, which ensure optimal symmetry in the finished product. The upper mold includes four holes, and the upper mold is equipped with four guiding bars. The ease-release material is carefully applied to both molds to begin the process, facilitating subsequent removal of the shaped soft muscle without any trouble. The silicon material and the silicone curing agent are mixed precisely in a ratio of 100:2 g to obtain a homogeneous mixture. This well-balanced composition is carefully poured into the lower mold; the upper and lower molds are tightly closed and secured. The curing process involves exposing the mold to vacuum pressure in a vacuum oven while maintaining a temperature of 40°C. It also involves exposing it to pressure. The next step involves unsealing the upper and lower molds, which reveals the half-muscle, which can then easily be joined to its corresponding counterpart as shown in Figure 10.

**FIGURE 11:**
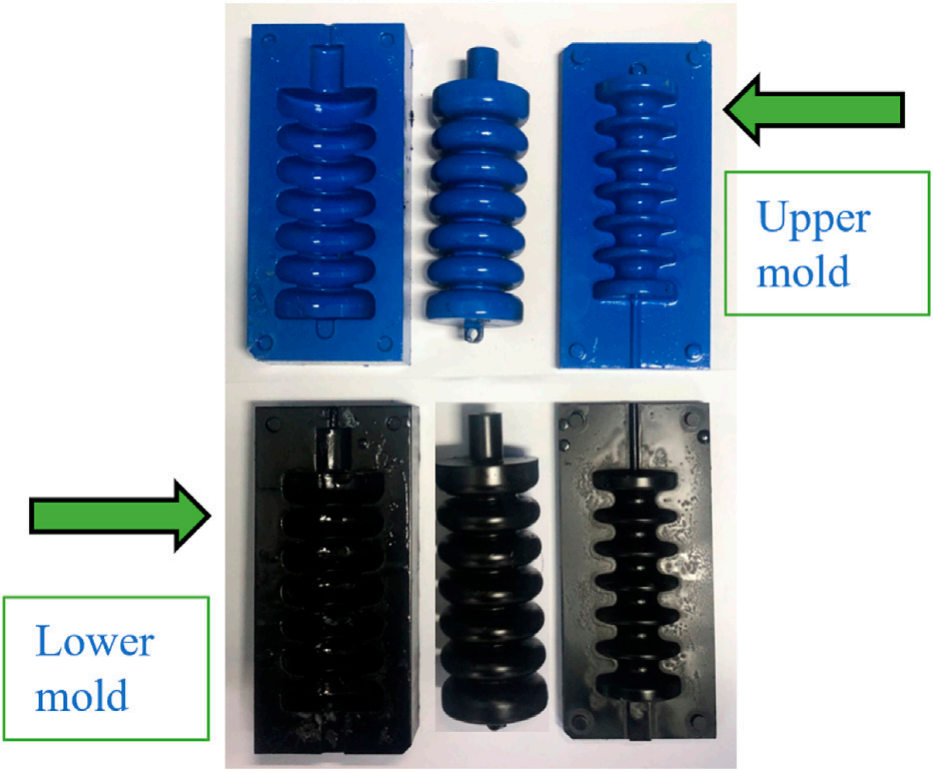
Demonstrates the mold components and the resulting soft muscles after extraction from the molds, as well as the process of adhering the two muscle halves together to form the complete soft muscle for ankle rehabilitation applications.

Based on previous experiments and empirical evidence, the most effective adhesive for bonding two silicone components was the silicone material itself, as shown in Figure 11. It was established that this is the silicon material itself. After successfully joining the soft muscle half, an intensive assessment is performed to determine if there are no air leaks. This assessment is made by submerging the joined halves in a water jar with varying air pressure and exposing them to water. This review ensures the integrity and reliability of the final soft muscle and confirms its suitability and effectiveness for ankle rehabilitation applications. It ensures the integrity and reliability of the final soft muscle.

## 6 Soft muscle experimental test

**FIGURE 12:**
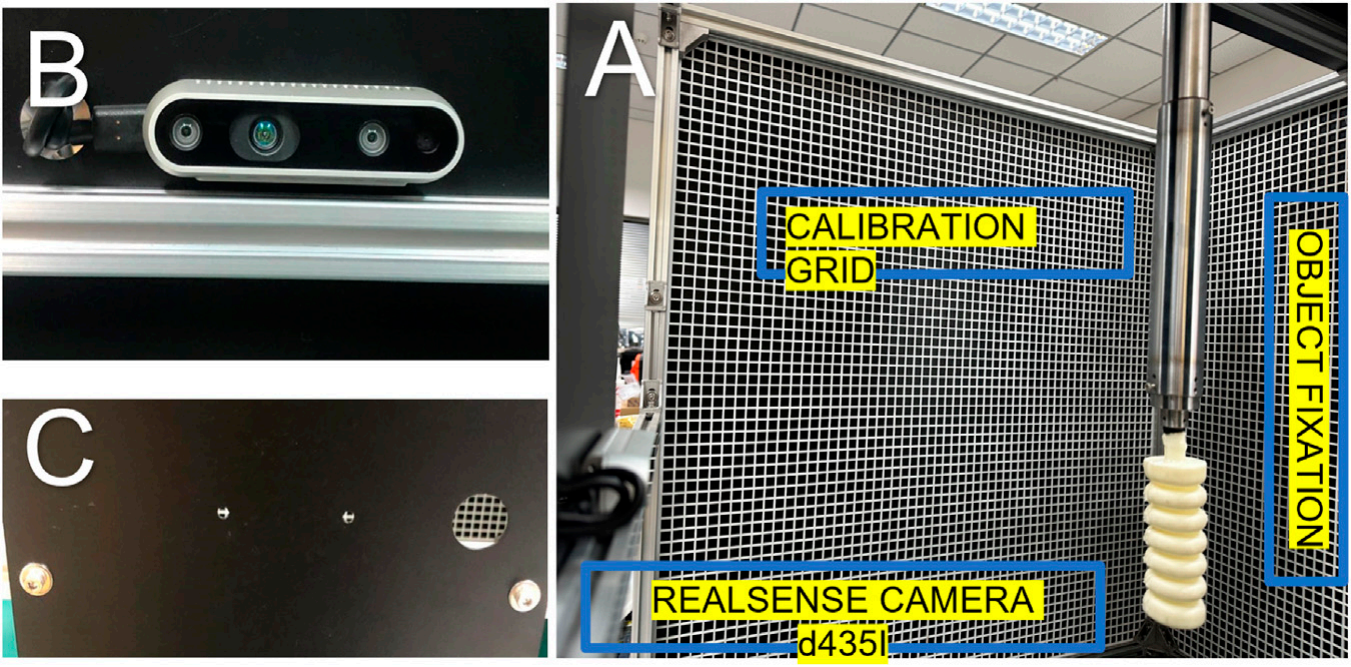
The experiment platform for measuring the elongation in the *Y*-axis regarding the pressure head change **(A)** The experiment space where the soft muscle fixed from one end in 3D space, **(B)** the real sense D435i camera detecting a video for the movement, and **(C)** the laser cut plate for camera fixation.

An experimental platform was developed and implemented with meticulous attention to detail to investigate the deformation of soft muscle during actuation with different pressures ranging from 100 to 145 (Kpa) for ankle rehabilitation. The experimental setup consisted of several critical components, each serving a specific purpose to ensure accurate and reliable results. The soft muscle was first securely fixed at one end in a 3D environment, allowing for unrestricted movement and deformation. This freedom of movement was essential for observing muscle behavior under different pressure conditions. To facilitate precise measurements of the muscle’s displacement, a 1 m × 1 m cubic cell was employed as the workspace, with a grid sheet positioned behind the muscle as a reference to ensure accurate displacement measurements throughout the experiment, as shown in Figure 12. The grid sheet was placed at a fixed distance behind the muscle, and its lines were in a contrasting color to the muscle to allow for easy identification of the muscle’s displacement.

To deliver pressurized air and secure the muscle in place, cantilever support was attached to the muscle and suspended at the top of the cubic cell. The cantilever support was designed to be dual-purpose, essential in securing the muscle and providing the necessary pressure head changes. The pneumatic system incorporated into the platform allowed for regulating the pressure and flow rates delivered to the muscle, enabling precise control over the muscle’s response to pressure head variations. Accurate capture of the muscle’s movement was essential for analyzing its behavior during the experiment. To accomplish this, a high-resolution RealSense D435i camera was employed to record video footage of the muscle’s displacement in the X, Y, and Z directions. The camera’s high resolution provided an accurate and comprehensive record of the muscle’s response under varying pressure conditions, which was synchronized with other experimental parameters using LabVIEW software. The camera was positioned at a fixed distance from the muscle. It was oriented perpendicular to the grid sheet to ensure accurate measurement of the muscle’s displacement in all three dimensions.

**FIGURE 13:**
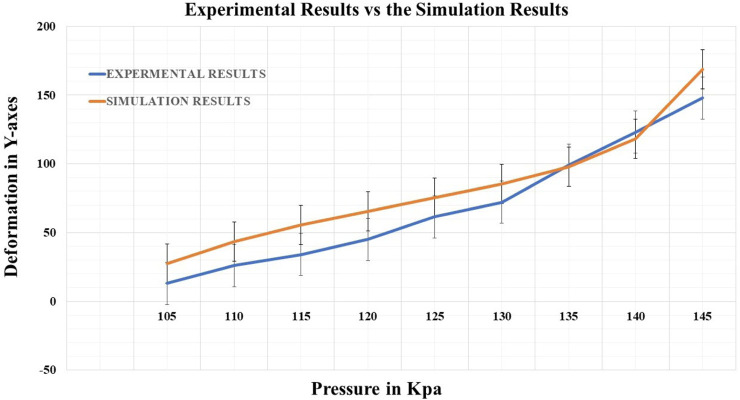
Demonstrates the correlation between the deformation in the *Y*-axis with respect to the change of the pressure in the Simulation compared to experimental tests.

Deep learning position estimation models were used to locate and measure the soft muscle end tip position x y z, marked with a red mark, to investigate the relationship between pressure head changes and soft muscle elongation for ankle rehabilitation. The algorithm defined displacement relative to the calibrated end tip’s first position. Before starting the measurement process, the end tip position was located using an initial calibration procedure. The observed position changes were then plotted with respect to the change of pressure, as shown in Figure 13. The figure shows the muscle’s original length and how it changes during different pressures, compared to the simulated results. The simulated data and experimental data match greatly, indicating the accuracy and reliability of the experimental platform and the position estimation models. Overall, this experimental work aimed to shed light on the complex relationship between pressure head changes and soft muscle elongation for ankle rehabilitation. The intricate design of the experimental platform, combined with the use of LabVIEW for data acquisition and control, facilitated precise control and measurement of the muscle’s response, establishing it as a valuable tool for delving into the mechanics of soft muscle systems. The results of this study are expected to contribute to a deeper understanding of the behavior of soft muscle systems under varying pressure conditions, with potential applications in fields such as bioengineering and biomechanics.

In the realm of artificial muscle systems research, a comprehensive comparison was conducted between McKibben artificial muscles, Festo pneumatics, and an actuator specially designed within this study. The evaluation focused on several critical parameters, including actuation speed, force generation, durability, ease of control, and fabrication. These factors are instrumental in determining the design, operation, and overall efficacy of artificial muscles, particularly in applications such as ankle rehabilitation.

Actuation speed was a key consideration. The actuator developed in this study was engineered to respond swiftly to control inputs, mirroring the rapid actuation characteristic of McKibben muscles. In contrast, slower actuation speeds were observed in Festo actuators, a trait shaped by their distinct design and operational principles. In terms of force generation, McKibben artificial muscles have been widely recognized for their high force-to-weight ratio. The actuator developed in this study, while not precisely matching the force generation of McKibben muscles, was found to generate a substantial force aptly suited for the intended applications. Festo actuators, conversely, were found to offer a range of force generation capabilities, primarily determined by the specific model, but a high force output is generally provided.

The durability of these artificial muscles was also examined. Both McKibben muscles and Festo actuators were found to exhibit robustness in a variety of applications. The actuator developed within this study, constructed from durable silicone and featuring an innovative design, also demonstrated promising durability, as evidenced by repeated testing cycles. Ease of control emerged as another vital parameter. The control of McKibben muscles can prove complex owing to their non-linear behavior. Festo actuators, despite being easier to control, often necessitate complex control systems. This complexity was mitigated in the developed actuator through design for easy control, a feature augmented by a LabVIEW-based control scheme.

Fabrication was another domain where the developed actuator excelled. It was designed with a strong focus on simple fabrication and assembly, as detailed in this paper. In contrast, both McKibben and Festo actuators were found to require more complex fabrication and assembly processes, which can pose significant challenges to their production and implementation.

Finally, McKibben artificial muscles and Festo pneumatic actuators each demonstrate their own strengths and have proven their effectiveness in various applications, the soft actuator developed in this study exhibits promising characteristics. These attributes position it as a potential candidate for targeted applications, particularly in the field of ankle rehabilitation.

## 7 Conclusion

In this research study, a soft pneumatic muscle was successfully developed and evaluated to replicate the intricate ankle motions necessary for effective rehabilitation, specifically emphasizing the crucial *x*-axis rotational movement during walking. The study achieved controlled expansion, accurate measurement, and comprehensive analysis of the muscle’s performance by integrating precise geometrical parameters, air pressure regulation, and advanced experimental techniques. Rigorous finite element simulation and experimental investigations provided compelling evidence of the soft muscle design’s adaptability, controllability, and effectiveness, establishing a solid foundation for future advancements in ankle rehabilitation and soft robotics. The experimental results revealed noteworthy findings. Under a pressure of 145 kPa, the soft muscle exhibited a substantial deformation along the *y*-axis (y-def) measuring 165 mm, emphasizing its significant elongation primarily in the *y*-direction. Conversely, the deformations along the *x*-axis and *z*-axis were minimal, measuring only 0.056 mm and 0.0376 mm, respectively, indicating negligible elongation along these axes. This observation underscores the specific and targeted response of the soft muscle to pressure actuation in the intended direction. Furthermore, the findings demonstrated the controllability of the muscle across different pressure values, showcasing an elongation range spanning from 0 mm to 165 mm. This remarkable adaptability and controllability enable precise regulation of the ankle’s rotational angle, accommodating the unique needs of individual patients and facilitating tailored rehabilitation protocols. The comprehensive understanding gained from this study significantly contributes to soft robotics and offers valuable guidance for future research and development in medical applications. The experimental platform developed for investigating the soft muscle’s deformation under various pressure conditions proved a reliable and accurate tool, enabling precise control and measurement of the muscle’s response. Overall, the results obtained from this study enhance our understanding of the intricate relationship between pressure head changes and soft muscle elongation for ankle rehabilitation. The measured values reinforce the performance and robustness of the soft muscle design, providing a solid foundation for further advancements in ankle rehabilitation and soft robotics. This research opens up new possibilities for improving ankle mobility and overall ambulatory function, leading to enhanced rehabilitation outcomes in clinical practice.

## Data Availability

The raw data supporting the conclusion of this article will be made available by the authors, without undue reservation.
